# High prevalence of atypical delayed enhancement in alkaptonuria

**DOI:** 10.1186/1532-429X-16-S1-P392

**Published:** 2014-01-16

**Authors:** Marcus Y Chen, Sujata M Shanbhag, W Patricia Bandettini, Peter Kellman, Vandana Sachdev, Andrew E Arai, Wendy J Introne

**Affiliations:** 1 National Heart, Lung and Blood Institute, National Institutes of Health, Bethesda, Maryland, USA; 2 National Human Genome Research Institute, National Institutes of Health, Bethesda, Maryland, USA

## Background

Alkaptonuria is a rare autosomal recessive metabolic disorder with an incidence of 1 case in 250,000 to 1 million live births. This genetic abnormality involves the tyrosine metabolism pathway which results in homogentisic acid accumulation throughout various tissues, including the heart. The purpose of this study is to prospectively determine the prevalence of cardiovascular abnormalities characterized by MRI in patients with alkaptonuria.

## Methods

A group of 57 consecutive adult patients with laboratory confirmed alkaptonuria were referred for evaluation including transthoracic echocardiography and cardiac MRI, which included cine and phase contrast imaging at either 1.5T or 3T. Phase-sensitive inversion-recovery delayed enhancement imaging was performed after gadolinium-DTPA contrast administration. Late gadolinium enhancement patterns were determined by consensus read between two experienced cardiologists using a 17-segment model.

## Results

Overall, 56 completed cine MRI, 53 received gadolinium contrast and 1 subject experienced claustrophobia precluding any imaging. Of the 56 who had CMR imaging, 77% (41 of 56) were male and the average age was 49 ± 12 years old. The prevalence of atypical delayed enhancement was 70% (37 of 53) and predominately involved the right ventricular insertion point (84%, 31 of 37 patients). Other delayed enhancement patterns included midwall (30%, 11 of 37), near aortic root (30%, 11 of 37), focal nodular (11%, 4 of 37), mitral papillary muscle involvement (8%, 3 of 37), and patchy/intermediate (5%, 2 of 37). Involvement of the right ventricular point has been associated with pulmonary hypertension; however, this group of alkaptonuria patients had normal estimated right ventricular systolic pressures measured on echocardiography performed within 1 day (median). The number of segments with atypical delayed enhancement was 2.7 ± 1.4 per patient. The prevalence of any atypical delayed enhancement trended higher for females vs. males (86% vs. 62%, p = NS) and was not associated with age. One subject had subendocardial delayed enhancement in a coronary artery distribution consistent with myocardial infarction.

## Conclusions

Myocardial fibrosis based on late gadolinium enhancement abnormalities, especially involving the right ventricle insertion site, are common in patients with alkaptonuria despite not having pulmonary hypertension or cardiac structural abnormalities and suggests myocardial involvement due to this metabolic disorder. Further study is required to determine the etiology and clinical significance.

## Funding

This work was funded by the Division of Intramural Research, National Heart, Lung, and Blood Institute, National Institutes of Health, USA.

